# A New Apparatus for Anesthesia with Ethyl Chloride

**Published:** 1919-01

**Authors:** 


**A New Apparatus for Anesthesia with Ethyl Chloride.** By MARCEL BUREAU. *La Presse Médicale*, September 23, 1918.

This apparatus is composed of a sort of gutter, a chamber or space (*c*) fixed to a bladder, and a mask similar to those in use in *Ombredanne*'s apparatus. A tube filled with ethyl chloride is introduced into the gutter. Its extremity protrudes into the space (*c*) and it is maintained in position by means of a screw. Space (*c*) surrounds the gutter on two sides; a tube for the admission of the air opens into it, and it has two apertures corresponding to the mask and the bladder. A valve commanded by an external lever closes the tube. It can be removed, however, thus allowing the ethyl chloride to flow out slowly, its vapors filling up the space (*c*) and the bladder.

*Use of the Apparatus.* The tube of ethyl chloride being previously opened, it is placed in the gutter. A simple pressure on the lever opens it and the ethyl chloride flows out. Anesthesia is obtained rapidly, and the patient does not suffer from suffocation or strong excitement. When anesthesia has been obtained, it is kept on as long as necessary by letting out of the tube, every half minute, 1/2 cc. of ethyl chloride.

This method has given good results in all sorts of operations, however long. Nevertheless, for laparatomies, anesthesia with ethyl chloride might prove insufficient and is not considered advisable.

